# Supplementary material for: Fast and accurate imputation of genotypes from noisy low-coverage sequencing data in bi-parental populations
Source: PLoS One. 2025 Jan 30;20(1):e0314759. doi: 10.1371/journal.pone.0314759 (PMC11781708; doi:10.1371/journal.pone.0314759)
Supplement: S1 Fig — (PDF) [file pone.0314759.s004.pdf]

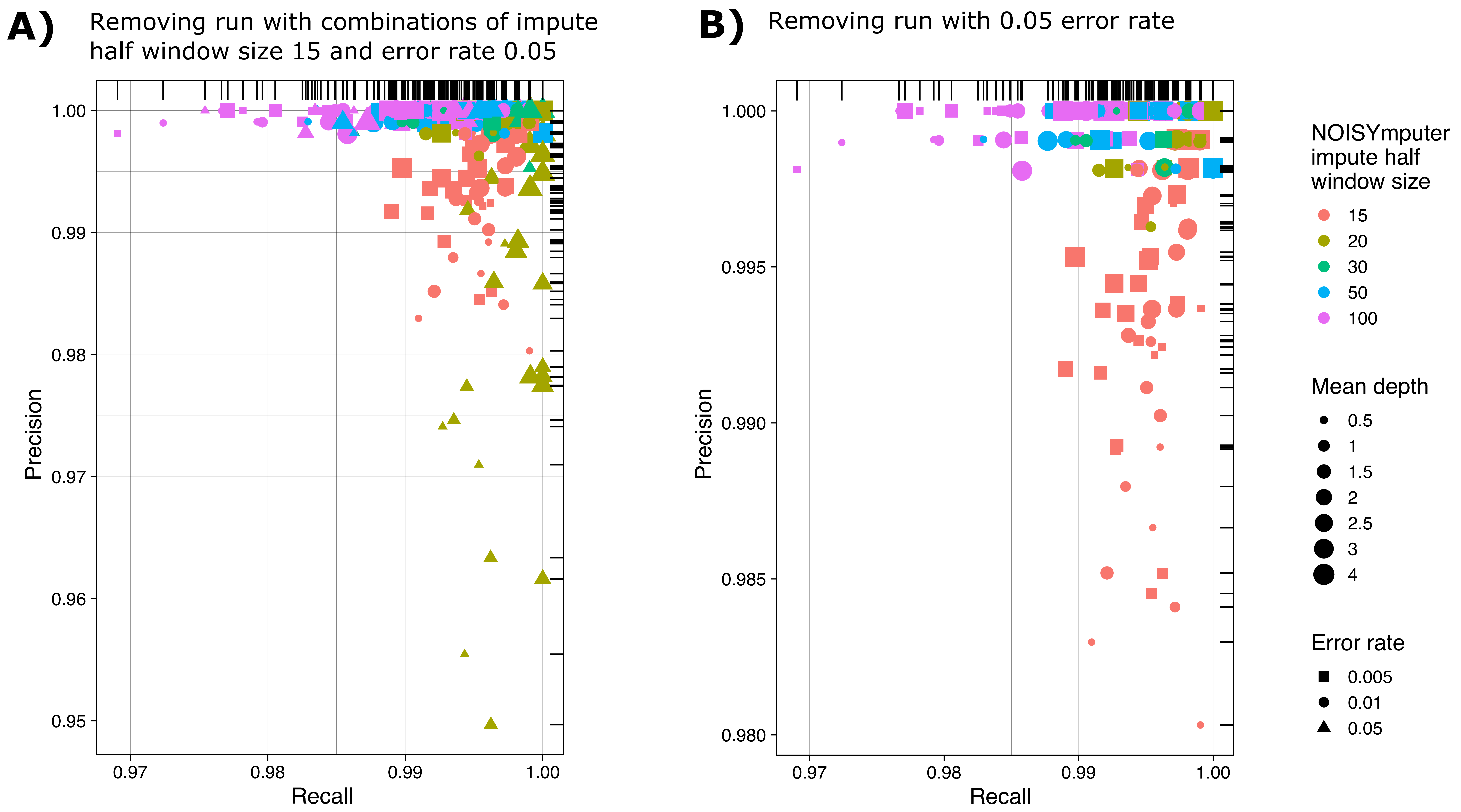

A)
Removing run with combinations of impute half window size 15 and error rate 0.05
B)
Removing run with 0.05 error rate
Precision
Recall
1.00
0.99
0.98
0.97
0.96
0.95
0.97
0.98
0.99
1.00
1.000
0.995
0.990
0.985
0.980
NOISYmputer impute half window size
15
20
30
50
100
Mean depth
0.5
1
1.5
2
2.5
3
4
Error rate
0.005
0.01
0.05
