# Supplementary material for: Fast and accurate imputation of genotypes from noisy low-coverage sequencing data in bi-parental populations
Source: PLoS One. 2025 Jan 30;20(1):e0314759. doi: 10.1371/journal.pone.0314759 (PMC11781708; doi:10.1371/journal.pone.0314759)
Supplement: Attachment — Submitted filename: Response to Reviewers.pdf [file pone.0314759.s010.pdf]

PLOS ONE – PONE-D-24-15414

Manuscript: “Fast and accurate imputation of genotypes from noisy low-coverage sequencing data in bi-parental populations”

Response to Reviewers

Editor’s comments

1. Please ensure that your manuscript meets PLOS ONE's style requirements, including those for file naming. The PLOS ONE style templates can be found at https://journals.plos.org/plosone/s/file?id=wjVg/PLOSOne_formatting_sample_main_body.pdf and https://journals.plos.org/plosone/s/file?id=ba62/PLOSOne_formatting_sample_title_authors_affiliations.pdf

We carefully checked the styles requirements, and hope everything is fine.

2. Please update your submission to use the PLOS LaTeX template. The template and more information on our requirements for LaTeX submissions can be found at http://journals.plos.org/plosone/s/latex.

We updated the manuscript using the PLOS LaTeX template.

3. Thank you for stating the following financial disclosure:
"The French ANR project "LANDSREC" 385 (ANR-21-CE20-0012-03), the French Government France Génomique program through its International RIce 485 Genome INitiative “IRIGIN” project, and the CGIAR Research Program “RICE”"

Please state what role the funders took in the study. If the funders had no role, please state: ""The funders had no role in study design, data collection and analysis, decision to publish, or preparation of the manuscript.""
If this statement is not correct you must amend it as needed.
Please include this amended Role of Funder statement in your cover letter; we will change the online submission form on your behalf.

We included the amended role of funders in the cover letter.

4. Thank you for stating the following in the Acknowledgments Section of your manuscript:
"We thank Karine Labadie (CEA, Institut de Génomique, Genoscope, Evry, France) for sharing the WGS data for
the sequencing of rice populations, Christine Tranchant-Dubreuil (IRD, Montpellier, France) for her help with
retrieving the Rice_WGS data and François Sabot (IRD, Montpellier, France) for coordinating the IRIGIN
project. We are grateful to the Institut Français de Bioinformatique (IFB) for providing computing resources.
We also thank the Yale Center for Research Computing for guidance and use of the research computing
infrastructure. The following programs supported parts of this initiative: the French ANR project "LANDSREC"
(ANR-21-CE20-0012-03), the French Government France Génomique program through its International RIce
Genome INitiative “IRIGIN” project, and the CGIAR Research Program “RICE”."

We note that you have provided funding information that is not currently declared in your Funding Statement. However, funding information should not appear in the Acknowledgments section or other areas of your manuscript. We will only publish funding information present in the Funding Statement section of the online submission form.
Please remove any funding-related text from the manuscript and let us know how you would like to update your Funding Statement. Currently, your Funding Statement reads as follows:
"The French ANR project "LANDSREC" 385 (ANR-21-CE20-0012-03), , the French Government France Génomique program through its International RIce 485 Genome INitiative "IRIGIN" project, and the CGIAR Research Program "RICE""

Please include your amended statements within your cover letter; we will change the online submission form on your behalf.

We included the amended statement in the cover letter. Thank you for the help with this.

5. When completing the data availability statement of the submission form, you indicated that you will make your data available on acceptance. We strongly recommend all authors decide on a data sharing plan before acceptance, as the process can be lengthy and hold up publication timelines. Please note that, though access restrictions are acceptable now, your entire data will need to be made freely accessible if your manuscript is accepted for publication. This policy applies to all data except where public deposition would breach compliance with the protocol approved by your research ethics board. If you are unable to adhere to our open data policy, please kindly revise your statement to explain your reasoning and we will seek the editor's input on an exemption. Please be assured that, once you have provided your new statement, the assessment of your exemption will not hold up the peer review process.

All data were made available to download as required.

6. Please include captions for your Supporting Information files at the end of your manuscript, and update any in-text citations to match accordingly. Please see our Supporting Information guidelines for more information: http://journals.plos.org/plosone/s/supporting-information.

This was done.

The reviewers' comments pointed out that a revision is required to improve the current version of the manuscript. One of the most critical concerns regards the filtering step. It also needs to be clarified if the input of the three tested tools is the same. Please refer to the reviewers' reports and the Reviewer's Responses to Questions section for detailed comments, which could help you improve your manuscript. Please carefully address (and reply to) all the comments raised by all reviewers (this is mandatory).

We carefully addressed all the points raised by the Reviewers.

Reviewer #1

Note: all line numbers refer to the file "Revised Manuscript with Track Changes"

The authors propose a method to genotype bi-parental populations with low-coverage NGS technology and compare their approach against FSFhap and LB-Impute. An MLE algorithm as compared to HMM makes sense with the assumptions of the bi-parental populations with inbred and exclusively homozygous parental lines and the low running time and memory consumption looks good. However, I have some comments/questions as given below:

Major Comments

- There is a heavy filtering of SNPs before being used by the tool. Filtering multiallelic SNPs and small indels is common in genomic tools (but newer versions of tools are updating their pipelines to incorporate them now) but further filtering of SNPs based on HWE parameters, read counts, etc might be a bit excessive. Do you have any explanation about this?

Thank you for discussing the filtering steps. We do not filter on HWE – since we treat bi-parental populations only – but advanced filtering - before and after imputation - together with MLE-based imputation, is indeed a major feature of NOISYmputer. We filter on genotypic frequencies because some SNPs, generally those which come from erroneous mapping, produce segregations that depart heavily from what is expected in a bi-parental population, even in case of segregation distortion. Keeping those SNPs would add confusion, reducing the difference between the likelihoods of heterozygous and homozygous states.

The filter on read counts is particularly useful to eliminate SNPs that lay in repeated regions. Keeping them would lead to similar confusion. Furthermore, this is a flexible filter since it is applied per individual, and only the sites whose coverage is at least 10x higher than the average depth (calculated across all sites for the individual) are removed.

The thresholds of these various filters therefore depend on the dataset used and are recalculated to optimally filter the input VCFs. Thus, these "pre-imputation" filters should not be considered as a step preceding NOISYmputer, but as an integral part of the software, contributing to the quality of the imputation results.

Regarding multiallelic sites, they do not correspond to the case of crosses between pure lines, where only two alleles can segregate. This is why they are filtered out.

- How many SNPs are removed through these filtering criteria (in terms of percentage of all SNPs)?

We produced a table that we added as Supplemental S5_Table to better inform about the consequences of these filters on the number of remaining sites based on the real datasets analyses and modified main text (lines 423-426) to refer to it.
However, we would like to emphasize that the number of filtered sites heavily depends on the filters that may have been applied during the production of the initial VCF or the sequencing method. Thus, the user might feel that a large number of sites have been lost during NOISYmputer pre-imputation filtering steps if the initial VCF was minimally filtered beforehand and contained noise.

We strongly recommend applying as few arbitrary filters as possible when calling SNPs and producing the initial VCF. For example, in the Snakemake pipeline we provide, we only filter for biallelic sites, sites with at most 80% missing data across the entire population, and sites for which parents are polymorphic and homozygous. These filters are actually applied by NOISYmputer anyway, so applying them beforehand simply allows us to optimize the VCF storage by reducing their final size. No filters on depth, deviation from HWE, or other criteria are applied before running NOISYmputer.

- Do you compare NOISYmputer to FSFhap and LB-Impute with the input of these filtered variants to all three tools or are the filtering criteria for the three tools different? If variants are filtered differently for the three methods, is it possible to run the three tools on one VCF and compare the results? Is it possible that the high accuracy, better map size estimation, and breakpoint precision of the tool are the outcome of the heavily filtered set of SNPs?

It is correct that NOISYmputer does run filters that FSFhap or LB-Impute do not run, since NOISYMputer's algorithm is a combination of filtering and imputation. We thus ran again FSFhap and LB-Impute on the real rice and maize datasets pre-filtered by NOISYmputer, and, although the two programs produced better results than without the filtering, they still produced noisy imputed data and therefore extremely long genetic maps. We added Supplementary S3 Table to reflect these new analyses, and modified the text (lines 378-381). We did not re-test FSFhap on simulated data, because the program PopSimul does not produce SNPs with excess counts or incoherent genotype frequencies.

- Line 259: How do you define too many or too few? Is it based on the average read depth or a constant value of X?
Also it is possible that the SNPs with too many reads might be duplicated regions but this information seems to be lost when they are removed. Any comments on this?

Thank you for the question. It does indeed require some additional explanations. This parameter is not fixed, it varies depending on the dataset used. By default, when reading the input VCF file, if the depth of a site for a sample is 10 times greater than the average depth for that sample (across all its sites), the genotype at that site is set to a missing data. Also by default, no filter is applied on the minimum number of reads required to consider a site in a sample.

We added an explanation (lines 205-209) in the main text to clarify this section.

This filter is relatively "soft". For example it corrects only 0.02% of all genotypes as missing data in the Rice F2 dataset (9626/44845342) and 0.002% in the Maize F2 dataset (22/971901).

Regarding the second part of the question, we intentionally filter sites with excessive coverage relative to the sample's average coverage, as it might indicate the presence of (highly) duplicated regions. SNPs from duplicated regions are not informative because we cannot determine which alleles belong to which copy, making it impossible to compare haplotypes to estimate breakpoint positions.

We have also included this information in the standard output of NOISYmputer and added an output file that now reports the number of sites per sample (datapoints) set as missing data. This feature is currently available on the development branch of our GitLab repository and will be included in the next release of NOISYmputer.

- What is the reason that windows in this work are based on the number of variant positions around a central SNP and not base pair distance-based? More clearly, why is an m-window around SNPj defined as SNPj-m to SNPj+m and not all the variants whose base pair distance is less than m from SNPj. Most references have gaps in their sequences and the existing window-system in the manuscript will include variants which can be large distances away from the central SNP if such a gap exists between the two variants.

Why was such a window-system used instead of a base pair distance-based?

This is a very interesting question. We chose to use windows based on the number of variant sites - just like FSFhap or LB-Impute do - because the sites are far from being evenly dispersed on the genome. Thus, in base pair-based windows, the number of sites can vary greatly. Since the goal of the windows is to calculate likelihoods, which values depend on the number of sites in the window, using base pair-based windows would produce likelihood values that are difficult to interpret, especially when the number of sites is low. Also, the observed likelihoods depend on the real genotypes, which are modeled by recombination that is only faintly correlated with physical distance. Since the final goal is to calculate accurate genetic maps, in centimorgans, using physical distances do not seem to be appropriate.

Furthermore, we verified that the site-based method still produces good results when encountering breakpoints in low marker density regions (as shown in the simulated data in main text or in the table below). We looked at the top 25% of largest loose support intervals (which implied that sites are spaced apart) in the pseudo 3X samples of the Rice F2 dataset and observed that the distance to the true position of the breakpoint (in number of SNPs) is the same as in the complete dataset. Finally, even if the physical position in base-pair is higher on average, the median stays very reasonable and the drive of the mean is actually due to a single breakpoint among the 23 analyzed.

Lastly, we report several measures in the breakpoints output file for the user to filter the breakpoint results if needed. For example, we report the size of the loose support interval along with a proxy of a confidence interval, the number of SNPs analyzed and the number of SNPs that have data in the analyzed sample. See the Breakpoints.csv file in NOISYmputer output.

- Why was the comparison done with FSFhap and also not with FILLIN (as the main publication of FSFhap does)? FILLIN has imputation capabilities that can also be compared with the imputation done by NOISYmputer.

FILLIN, like Beagle or Impute2, was developed to impute genotypes in diversity panels (which the authors call inbred lines libraries). FSFhap, like NOISYmputer, was developed specially to address the problem of bi-parental populations. The authors themselves report that "FSFHap and FILLIN performed very similarly, but FSFHap imputed more heterozygous sites with increased accuracy" in the case of bi-parental populations. We thus considered that FSFhap is well more adapted for comparison with NOISYmputer.

- I could not find a direct link to the simulated and real dataset used in the experiments. Each dataset used in the experiments should be stated clearly with a proper link. For simulations, an open source repository (e.g., Zenodo) might be used.

We have made both real and simulated datasets available.

The simulated datasets can be reached at https://zenodo.org/records/13381283, while the real dataset is available at the IRD Dataverse: https://doi.org/10.23708/8FXUNC.

We added this in the main text line ~283 and line ~361.

Minor Comments

- Figure 2 shows that the performance of the algorithm is poor for data with high error rates. So even if the correct error rates are estimated, it does not help in generating accurate results and the tool depends on the underlying error rates. This is not a very big issue since the tool primarily deals with Illumina reads, which have very low error rates (as stated in lines 354-357). But future ideas related to extending these tools to error-prone long reads will be unsuccessful based on these results. Is it possible to generalize the model further to allow high error rates?

Indeed, higher error rates can impact the quality of NOISYmputer's results if the window size is suboptimal. However, correctly re-estimating these error rates allows for better adjustment of the genotypic and allelic filtering thresholds, as well as incoherent site detection and adjustment of the loose support interval where the recombination breakpoint is identified. This allows NOISYmputer to obtain overall good results even with high error rate and sub-optimal settings for window size (minimum accuracy of >72% and detection power of > 96%). Increasing the window size significantly enhances the robustness of NOISYmputer results though, even with error rates of 5%. For instance, in the simulated data (Figure 2), a window size of 30 achieves a minimum detection accuracy of 96% and a detection power of 99%, regardless of marker density or depth, even with a 5% error rate.

We are therefore confident that this model could be applied to long-read datasets with such high error rates in the future, as long as the window size is coherent with the dataset specificities.

We modified the text in the article (lines 317-320) and added Supplementary S1_Table to better explain the high error rate case.

Finally, we would like to underline the fact that FSFHap handles poorly high error rates (5%,c.f. Supplementary S1_Table), as it produces aberrant results (i.e., extremely long genetic maps).

- I would expect to see some stats about the performance of the algorithm in the abstract.

It's true we forgot to mention it. We added a few lines about this in the abstract.

- Line 48 states that "Reducing sequencing costs through minimized per-sample coverage has an important experimental downside: LC-NGS mechanically introduces a series of issues, the main ones being:"
However, I can't see any connection of issue 3 listed in line 60 with the data being low coverage. It's a problem with short-read sequencing.

We agree this is a general problem of short-read sequencing, not restrained to low-coverage.

We better separated general problems due to illumina short reads to problems proper to the use of Low coverage by splitting it in two sections (line 36).

- Line 117: Where does the equation for the proportion of the genome with no recombination come from? Reference?

We added an explanation for this equation in the Supplementary S2_File.

- Line 123: "computation time, which increases linearly according to the diplotype size" - Is there any time complexity analysis given as part of the work to show this?

We did not perform a proper complexity analysis. However, we did observe a linear relationship between execution time and window size. This is explained by the fact that the log-likelihood of a diplotype is the sum of individual values for each site. We added a sentence to explain why we claim that the complexity is O(n) (lines 113-114).

- Line 153-155: Are nAi and nBi numbers or is it a multiplication between n and Ai and Bi. If they are numbers, consider superscripting the A and B. It is not clear how they are being obtained. The binomial expansion has them as variables and their values can range from 0 to ni. But in Line 162 in the equation, they are no longer variables on the LHS of the equation while they exist on the RHS of the equation. Which value is chosen for nAi and nBi and how are they chosen?

We changed the A and B to subscripts, as superscript was a bit confusing to read.

nAi value corresponds to the number of A reads at site i, nBi value corresponds to the number of B reads at this same site i and ni is the total number of reads (depth) at site i (ni = nAi + nBi). Thus, for a given site, the values of A and B reads are known.

For example, if at position 255 in the genome for a sample there are 2 A reads and 0 B reads then nAi = 2, nBi = 0 and ni = 2. As we already know the probability of observing A reads under the three possible genotypes ($p(O_A | AA)$, $p(O_A | BB)$ and $p(O_A | AB)$), we use the binomial distribution to determine the probability of observing 2 A reads and 0 B reads at position 255 if the genotype was to be AA, BB or AB.

We added additional explanations in this paragraph, hopefully making it easier to follow.

- Line 210-225: If BB is the segment to the left of position j and AB is to the right, why is the BB window defined for j to j+m and vice versa for the AB window? Since BB is to the left, the window is for j-m to j.

This is correct, thank you for pointing out this error. We corrected the text properly.

Further definition of PSI as a product of 1-probabilities for the two regions. There is no explanation on why this approach has been taken.

As illustrated in the following example: $p(Wj = BB)$ and $p(Wj = AB)$ values are close to 1 outside the crossover region, while their values drop rapidly, close to zero, when getting close to the crossover. Thus, the values 1 - $p(Wj = BB)$ and 1 - $p(Wj = AB)$ are close to 1 nearby the crossover.

An effective way to guarantee that the two values 1 - $p(Wj = BB)$ and 1 - $p(Wj = AB)$ are significantly superior to zero – and thus that the crossover is located closeby – is that their product PSI is significantly greater than zero. We can take a conservative threshold for PSI – 0.05 for instance – to guarantee that the subsequent searching region will cover the crossover position with a high probability.

We added an explanation for this method in the Supplemental S3_File and referred to it line 171.

- Line 244-245: The formulation of homozygous to heterozygous transitions seems to be easily generalizable for double recombination events of transitioning from homozygous to homozygous. Why not allow such events as default and allow users to choose whether to consider the double recombination transitions?

We completely agree. We are currently working on modeling double recombination events to compare their likelihoods to non-recombination events, in order to allow the detection of closely located crossovers. However, we were planning to include this feature in the next version of NOISYmputer and do not feel that we have enough time to implement it properly in version 1. This is commented in the second paragraph of the final "Suggestions for improvement" section.

- Line 320: Does PoPSimul also simulate gaps in the reference or is it a uniform distribution of SNPs?

PopSimul does not simulate gaps in the reference genome. It uses fixed SNP positions. The purpose of PopSimul is only to evaluate the performance of imputation under various levels of depth, sequencing error and missing data.

- Line 338-342: It can also be considered to report the results using other metrics like precision-recall or Matthews Correlation Co-efficient etc. Maybe even with a confusion matrix.

Thank you for the suggestion. In fact, the "detection power" and "detection accuracy" we used in the manuscript are exactly precision-recall measures. Thus, we modified the main text (along with the Figure 2, its legend and the supplementary S1_Fig, similar to Figure 2) to switch from "accuracy" and "power" to "precision" and "recall".

- Line 316-317: Specs of the cluster are given in the link but which of these machines did you use for the experiments. This information can also be added as a single line regarding the specs of the machine used.

As the computing facility we used consists of a cluster of several machines from a national platform, we added an explanation and detailed description of the machine specs in the Supplementary S1_file and we refer to it in the main text (lines 272-273) .

Typos

- Line 102: "leaving unimputed the regions"

corrected

- Line 132: “once could set”

corrected

- Line 157-159: End bracket missing in the expression in the middle.

corrected

- Line 218: Should this be SNPj+m and SNPj-m respectively, where “m” is subscripted?

yes it should, we corrected the typo.

Reviewer #2

Note: all line numbers refer to the file “Revised Manuscript with Track Changes”

The authors propose a specific scheme for handling imputation of individuals where the original genetic stock come from two completely inbred lines. They propose to compute the total number of reads supporting the two generalized alleles (A and B) and make the primary imputation decisions based on these counts. They claim that this technique will be more tolerant to sequencing and mapping errors compared to other prevalent approaches.

The authors claim that this approach is more efficient than a Hidden Markov Model, but in my mind, what's proposed is similar to a HMM where the emitted symbols are the read counts in each window. Combining windowing with transition probabilities for long range haplotype consistency wouldn't be impossible.

Thank you for this comment. Actually, what makes NOISYmputer more efficient is the combination of advanced filtering and windowed imputation. We agree that it would be possible to combine our windowing approach with transition probabilities. However, we are unable to include transition probabilities in the current version of the algorithm, since it would require a very long process of implementation, testing and re-writing large parts of the manuscript. Also, FSFhap and LB-Impute, which are based on transition probabilities, provide poor results compared with NOISYmputer, so we believe that the maximum-likelihood method used alone might be more robust.

I would also be somewhat weary against the argument that it makes sense to have a single global error rate per origin line, since mapping and sequencing errors are highly variant-specific. On the other hand, I can of course agree that two parameters would maybe be better than a single global epsilon.

We agree with this comment. Indeed, there is a program, GBScleanR, that estimates local error rates along the genome. However, when we tested it, it produced even more noisy results than FSFhap and LB-Impute, and that is why we decided to discard it from our benchmarking tests. But this does not mean that NOISYmputer could not benefit from local error estimation. We added a comment on this in the fourth paragraph of the Suggestions for improvement section (lines 555-560).

The use of the reconstructed chromosome length as a metric for quality is ingenious, but it takes some time to wrap your head around it.

Thank you for pointing this out. The essence of the method is explained in the text:

"Conversely, datasets with a high rate of genotyping errors will exhibit map expansion, resulting in a longer genetic map due to falsely imputed recombination breakpoints."

We hope this sentence is clear enough to the reader.

Overall, I find the contribution to be interesting, but there are several assumptions made on the type of dataset and I think the manuscript would benefit from more clearly spelling these out and also contrast to related scenarios where they would not be applicable.

The current version of the manuscript describes dataset specification in terms of population type and input format files in the section "Data specifications". If this is not detailed enough, we'd be grateful if Reviewer 2 could provide more details on the kind of clarification needed.

Reviewer #3

The paper is written well, I understood the problem of imputation and authors' approach to the maximum likelihood estimation. The results provided are understandable. However, related work or literature review could be improved.

We could agree with Reviewer #3 on literature review and related work. However, we did our best to perform a careful and complete review of alternative algorithms and software for imputation of bi-parental populations. Should the Reviewer provide information on work(s) that we missed, we are happy to include it (them) in the manuscript.
