# Supplementary material for: Fast and accurate imputation of genotypes from noisy low-coverage sequencing data in bi-parental populations
Source: PLoS One. 2025 Jan 30;20(1):e0314759. doi: 10.1371/journal.pone.0314759 (PMC11781708; doi:10.1371/journal.pone.0314759)
Supplement: Attachment — Submitted filename: Response to Reviewers revision 2.pdf [file pone.0314759.s011.pdf]

PLOS ONE – PONE-D-24-15414

Manuscript: “Fast and accurate imputation of genotypes from noisy low-coverage sequencing data in bi-parental populations”

Response to Reviewers

Editor’s comments

The authors addressed the main Reviewers’ comments; however, a couple of concerns remain that must be considered. For instance, I agree with Reviewer 2 that the authors should improve the repository documentation. Please consider the two minor comments from Reviewer 1.

> Thanks very much for these new comments. We have addressed the different points raised and list them hereby.

Reviewer #1

Note: all line numbers refer to the file “Revised Manuscript with Track Changes”

• I think this sentence in the abstract has issues, it should be rephrased: "NOISYmputer is particularly convincing when comparing the genetic map obtained on real datasets as it estimates accurately the size of the map where the two other software can mistake from hundred to hundred thousands centimorgans."

> We agree that this phrase was unclear. We have rephrased it as:

“NOISYmputer consistently provides precise map size estimations when applied to real datasets while alternative tools may exhibit errors ranging from 3 to 1845 times the real size of the chromosomes in centimorgans.”

• Link to data in Zenodo should be added under "Availability"

> We added the data link in the Availability section

• The code repository will benefit from better documentation about the list of output files that are generated. Multiple output and intermediate files are generated but no explanation for what each file represents have been provided.

> We added documentation on every file generated by NOISYmputer in the code repository. It is available at

https://gitlab.cirad.fr/noisymputer/noisymputerstandalone/-/blob/dev/documentation.md?ref_type=heads

Reviewer #2

• I still think there would be room for improvement in the presentation, and I can agree with the request from reviewer 3 for additional references. I think even tools such as R/QTL and the original Lander-Botstein model are relevant in the sense that inferring trait status and outright imputation are mirror problems, especially in the biparental case.

> Although we agree that inferring trait status and genotype imputation are mirror problems, in the case we treat, only the genotypes are available. This is why we do not refer to the works of Lander & Botstein (Mapmaker/QTL) or Karl Broman (R/QTL). Also, the methods implemented by Mapmaker or R/QTL can only treat arrays made of genotype datapoints, while NOISYmputer takes the (Illumina or similar) short reads counts to calculate genotype probabilities, so these previous methods do not use the full information provided by sequencing-based genotyping. We hope this clarifies why we do not refer to these – or similar – methods.
